# Source/sink interactions underpin crop yield: the case for trehalose 6-phosphate/SnRK1 in improvement of wheat

**DOI:** 10.3389/fpls.2014.00418

**Published:** 2014-08-25

**Authors:** David W. Lawlor, Matthew J. Paul

**Affiliations:** Plant Biology and Crop Science, Rothamsted ResearchHarpenden, UK

**Keywords:** source/sink, wheat, grain yield, yield components, food security, sucrose, trehalose 6-phosphate, SnRK1

## Abstract

Considerable interest has been evoked by the analysis of the regulatory pathway in carbohydrate metabolism and cell growth involving the non-reducing disaccharide trehalose (TRE). TRE is at small concentrations in mesophytes such as *Arabidopsis thaliana* and *Triticum aestivum*, excluding a role in osmoregulation once suggested for it. Studies of TRE metabolism, and genetic modification of it, have shown a very wide and more important role of the pathway in regulation of many processes in development, growth, and photosynthesis. It has now been established that rather than TRE, it is trehalose 6-phosphate (T6P) which has such profound effects. T6P is the intermediary in TRE synthesis formed from glucose-6-phosphate and UDP-glucose, derived from sucrose, by the action of trehalose phosphate synthase. The concentration of T6P is determined both by the rate of synthesis, which depends on the sucrose concentration, and also by the rate of breakdown by trehalose-6-phosphate phosphatase which produces TRE. Changing T6P concentrations by genetically modifying the enzymes of synthesis and breakdown has altered photosynthesis, sugar metabolism, growth, and development which affect responses to, and recovery from, environmental factors. Many of the effects of T6P on metabolism and growth occur via the interaction of T6P with the SnRK1 protein kinase system. T6P inhibits the activity of SnRK1, which de-represses genes encoding proteins involved in anabolism. Consequently, a large concentration of sucrose increases T6P and thereby inhibits SnRK1, so stimulating growth of cells and their metabolic activity. The T6P/SnRK1 mechanism offers an important new view of how the distribution of assimilates to organs, such as developing grains in cereal plants, is achieved. This review briefly summarizes the factors determining, and limiting, yield of wheat (particularly mass/grain which is highly conserved) and considers how T6P/SnRK1 might function to determine grain yield and might be altered to increase them. Increasing the potential rate of filling and mass/grain are ways in which total crop yield could be increased with good husbandry which maintains crop assimilation Cereal yields globally are not increasing, despite the greater production required to meet human demand. Careful targeting of T6P is showing much promise for optimization of source/sink for yield improvement and offers yet further possibilities for increasing sink demand and grain size in wheat.

## INTRODUCTION

Understanding of the integration of the processes of carbon assimilation and of growth of organs in higher plants is advancing rapidly, but mechanisms are complex and incompletely understood. One mechanism linking the two has been identified: it involves trehalose 6-phosphate, the precursor of trehalose acting as a sensor of sucrose concentration (Paul, 2007; Paul et al., 2008), which regulates the activity of a protein kinase SnRK1: this activates (de-represses) or represses gene expression for proteins of basic metabolism. Although T6P and TRE usually occur at very small concentrations compared with many other carbohydrates, T6P increases substantially – albeit over the micromolar range – with greater availability of sucrose and larger fluxes of sucrose to organs and correlates strongly with changes in carbohydrates, e.g., starch deposition in the endosperm of wheat grains is associated with T6P in the endosperm of wheat (Martínez-Barajas et al., 2011). It is now very clear that T6P is a signal of sucrose availability with large effects throughout metabolism (Nunes et al., 2013a; Lunn et al., 2014). The ratio of T6P/sucrose is very constant in tissues but manipulation of the enzymes of synthesis and breakdown of T6P may have profound effects on the ratio which then affects many processes in plants (van Dijken et al., 2004; Yadav et al., 2014). The effects of altering T6P on plant development and growth have been well demonstrated by genetic modification. Frequently development of shoot, leaf, and root is altered, giving smaller, thicker organs when T6P exceeds its normal concentration range: leaves are often darker green indicating more chlorophyll per unit area (Iordachescu and Imai, 2008) which increases the photosynthetic capacity (Pellny et al., 2004). Decreases in T6P result in the opposite phenotype, where leaves are paler, larger, and thinner (Pellny et al., 2004). Such changes in vegetative phenotype are reminiscent of sun and shade leaves respectively indicating that carbon, via T6P, has an input into leaf development as an indicator of energy and resource availability independent of the light environment. However, productivity/plant or of a crop (productivity/unit area of land surface) may not reflect the changes at the leaf level of organization because of the interaction of light interception and photosynthetic rate per unit leaf area (LA) and the total LA. T6P also plays an essential role in regulation of sugar-induced leaf senescence (Wingler et al., 2012), which affects LA and thus light interception and photosynthesis. The effects of altering T6P can be variable. For example, Yeo et al. (2000) constitutively expressed T6P synthase (TPS1) in potato plants which had severely retarded dwarf growth, altered leaf shape, and yellowing, together with aberrant roots. However, Han et al. (2005) made a similar transformation which did not affect growth: the reasons for such differences are likely that because T6P is such a powerful regulator subtle differences in amounts and distribution acting together with particular environmental conditions and stages of development can produce contrasting outcomes. A major consequence of modifying T6P is alteration of carbohydrate balance, with accumulation of carbohydrates, e.g., starch in potato tubers (Debast et al., 2011) and wheat grain (Martínez-Barajas et al., 2011), and profoundly alters growth (Delatte et al., 2011). Genetic transformation of rice with TPS and alteration of TRE concentration has been claimed to increase osmolyte concentrations and so confer “drought tolerance” (Garg et al., 2002; Jeong et al., 2010) but it is now recognized that this “drought tolerance” is a result of decreased growth and stomatal conductance, so slowing water loss and the onset of drought (Lawlor, 2013). These effects have been observed in different species, of different ages, and in different organs showing T6P to be a general, probably universal, regulator of plant carbohydrate metabolism, development, and growth. The importance of T6P has been supported by the multiplicity of genes coding for TPS and T6P phosphatase (TPP), 21 in *Arabidopsis thaliana*. This suggests multiple sites of action and likely different methods of regulation which affect many parts of cellular metabolism, function and growth (Zhang et al., 2009; Paul et al., 2010): altering TRE metabolism by genetic modification is recognized as a likely way to improve plant and crop performance (Almeida et al., 2007). T6P is now recognized as a signal molecule, with a central role in metabolism as a sensor of carbohydrate, specifically sucrose status. Coupling of sucrose concentration to UDP and G6P synthesis and thus to T6P concentration provides a sensor of increasing sucrose concentration and availability. For a comprehensive review see Paul et al. (2008). More recent aspects of trehalose metabolism and the effects on growth and carbohydrate partitioning are considered by Nunes et al. (2013a) and Lunn et al. (2014).

Many of these effects of T6P can be explained through its action (Schluepmann et al., 2003) as a regulator of the protein kinase system which is central to cell, organ and plant development, growth, and ultimately production of yield in relation to carbon, energy, abiotic, and biotic stresses (Halford and Hey, 2009). This allows the sucrose concentration to be tightly coupled in very specific ways via T6P to the kinase system. T6P inhibits the activity of the SNF1-related protein kinase, SnRK1. SnRK1 belongs to the SNF1/AMPK group of protein kinases and is the plant homolog of AMP-activated protein kinase in mammals, a sensor maintaining cellular energy homeostasis by regulating anabolic and catabolic processes and balance. This group of kinases, and other regulatory mechanisms such as the transcription factor bZIP11 with which they interact (O’Hara et al., 2013) regulate the cell cycle, cell division, apoptosis, and cell and tissue metabolism; modification of the system results in changed growth (Guérinier et al., 2013). Many proteins, such as sucrose phosphate synthetase and nitrate reductase, are inhibited by phosphorylation catalyzed by specific protein kinases and activated by de-phosphorylation. With increased sucrose concentration, T6P concentration rises, inhibiting SnRK1 so changing the balance of gene expression. This represses or de-represses protein synthesis in a highly coordinated manner and thus promotes synthetic (anabolic) processes, and represses breakdown (catabolic) processes culminating in greater growth. Increased growth requires, of course, synthesis of structural components such as cellulose and proteins for cell walls and therefore depends on the availability of sucrose and amino acids, etc., so can only occur under environmental conditions when carbon sources are available such as from active photosynthesis or during mobilization of reserves, e.g., during grain or tuber sprouting. However, specific modification of SnRK1 activity could allow more favorable allocation of resources to harvested organs when carbon is plentiful and better survival and recovery under conditions less favorable for growth. Targeting flexibility in the SnRK1 signaling and transduction system between these two states may enable improved crop performance (O’Hara et al., 2013).

**FIGURE 1:**
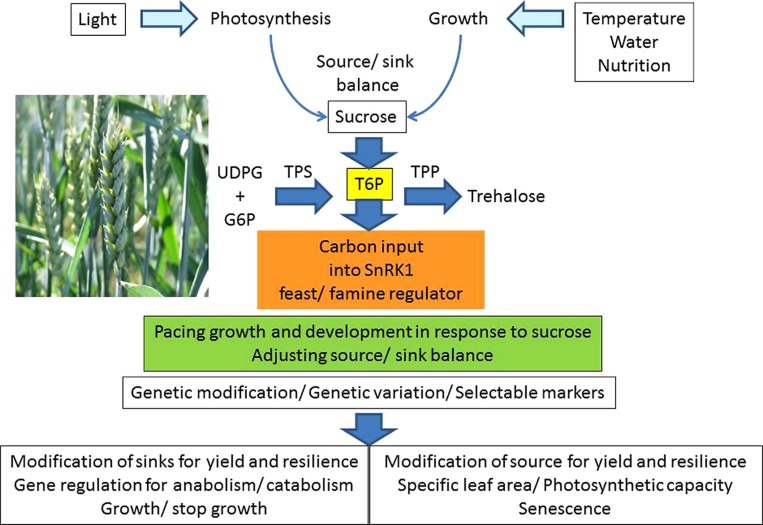
**Optimizing sucrose allocation for yield under variable environmental conditions**.

Protein kinases are also regulated by adenylate nucleotides and the adenylate kinase reaction. A large ATP concentration, and low ADP and AMP, signal conditions which stimulate anabolic metabolism with active growth. Under conditions which are not conducive to growth, such as when ATP synthesis is restricted in poor light, with inadequate inorganic phosphate supply or rapid water deficits (Lawlor and Tezara, 2009), the ATP/ADP decreases and the AMP concentration rises. These conditions activate the AMP kinases and stimulate phosphorylation of the target proteins, inactivating them. The SNF1-related kinases are a family of three groups (SnRK1, SnRK2, and SnRK3) with a total of 38 members in *Arabidopsis* (Halford and Hey, 2009). The implications for coordination of metabolism by interaction of sucrose concentration with T6P and SnRK1 with the kinase system are considerable (Jonak et al., 1999). This multiplicity of genes suggests, and indeed it is now widely accepted, that there are many inter-linked protein kinases which regulate gene expression and thus the protein synthesis of the cell. The kinases regulate metabolism, cell growth and development, and thus plant production. The kinase system, with T6P control of SnRK1 as part of a wider regulatory network is a very sensitive indicator of the state of the adenylates and carbohydrates which rapidly responds to the combined effects of environmental conditions and metabolism and integrates the longer-term responses. In addition there are complex feed-back and feed-forward steps which regulate the synthesis of TPS and TPP and thereby T6P synthesis (Zhang et al., 2009; Yadav et al., 2014). The T6P/SnRK1 system provides a mechanism which potentially underlies and explains many aspects of plant growth and yield formation (**Figure 1**). It might, therefore, be manipulated to increase yields of crops (Martínez-Barajas et al., 2011).

## BACKGROUND TO CROP PRODUCTION

It is important to consider the plant and crop “framework” on which agricultural crop production rests and by which the desired product – yield – is achieved. Detailed analysis of the production system in conjunction with current understanding of how trehalose metabolism interacts with cellular and organ development and growth may serve to identify where further analysis is needed and potential targets for over-coming production constraints. In addition the aim is to highlight the role of the environment, and the consequent crop–environment interaction, in production. Analysis of crop production, specifically of dry matter of vegetative or reproductive organs part of which may form the harvested economic yield, may be considered by taking a “crop-down” view. This indicates the nature of the overall processes which contribute to growth and may improve understanding leading to ways of altering the system and improving yield. Also, the ways that empirical changes to plant metabolism, such as those associated with genetic modifications by direct intervention in the genome, act to alter growth and production may be placed in the context of the larger-scale processes. This permits the biochemical events to be related to the crop. From this the underlying molecular mechanisms which ultimately determine the fluxes of material to developing and mature organs may be identified. Here the specific example of changes caused by altering T6P will be considered from this view-point.

In attempting to link crop production with biochemical events it is perhaps useful and important to highlight and consider the differences between the disciplines of agriculture and biochemistry in dealing with space and time. Biochemistry operates on short temporal and spatial scales. Agriculture is concerned with the growing season and production over large areas and integrates all the biochemical processes. Mathematical modeling of complex metabolic processes offers ways of examining such systems (Williams et al., 2010). Plant and crop physiology bridge the gap but relating small-scale processes to large scale in complex systems is difficult, because the details of the mechanisms are usually imperfectly understood, to which are added factors such as statistical variation in data and differences in conditions between the small-scale measurements (often made *in vitro* or on plants under non-field conditions) and those in the field. Also, the involvement of many, linked processes complicates analysis and often correlation between processes or events at the different scales is the best indication that a causal mechanism may be involved. However, analysis of the mechanisms is required if improvements in crop production are to result from detailed biochemical knowledge (Foulkes et al., 2011). Such understanding may also foster links to other disciplines, such as selection breeding of crops using quantitative trait loci (QTL) and molecular marker techniques (Kirigwi et al., 2007). As knowledge develops more quantitative systems analysis using mathematical and statistical procedures may follow. The following summarizes the agriculturally relevant features in crop production determining yield and then addresses how they are achieved and how yield may be increased by altering them. Then the role of the T6P/SnRK1 mechanism in relating assimilate production to yield will be addressed, together with how this system may be modified at the biochemical scale to alter plant growth and crop performance and increase yield.

## CROP PRODUCTION

The basic processes involved in crop production are very similar in all the staple crops – rice, wheat, maize, potato, etc. – which are short-term mono and dicot annuals, or have C3 or C4 photosynthesis. Here, for simplicity and its importance as a food source, wheat is taken as an example (see Frederick and Bauer, 1999; Slafer, 2007; Foulkes et al., 2011). Following germination of the seed which provides stored assimilates (starch, proteins) for initial growth of organs, the photosynthesizing leaves provide assimilates: amino acids for protein synthesis and sucrose as the carbohydrate. Sucrose is used in many processes, e.g., synthesis of cellulose for cell walls or ultimately consumed in respiration. There is a relatively long period of vegetative growth with development of secondary stems (tillers) and leaves, then a transition from vegetative to reproductive state, with formation of florets, ears, and finally grains. Grains fill with starch and protein during a relatively brief period and the seed matures. This constitutes the harvested yield which in all the cereals is predominantly composed of starch deposited in the grain, but with a very important storage protein component plus the protein in the embryo. At each stage the rate of supply of assimilates from the “source” leaves to the “sink” of growing or filling organ must meet the potential rate of growth or filling of the organ, if the maximum obtainable rate is to be achieved. Similarly, supply must meet demand if the potential size of the organ is to be attained. The “potential” rate of growth and size are genetically determined and upper limits to what can be achieved by the genetically based metabolic events in an environment which is optimum for all the processes. Detailed consideration of the subject is given by Slafer and Araus (2007), Araus et al. (2008), Cossani et al. (2010), Fischer and Edmeades (2010), and Reynolds et al. (2011). The ideal condition for yield production is optimization of all metabolic events with the environment. This includes optimizing rates of processes and also their duration. Note that the duration of developmental processes is generally determined by genetically based mechanisms which are often controlled by environmental factors. That is, the duration of processes is limited. If the rate of a process, such as the supply of assimilates to the grain, is sub-optimal (i.e., does not provide assimilates required for the “potential rate of growth”) the final size of the organ will be smaller than could be achieved if the “genetic potential” was fulfilled: full size cannot be achieved by simply extending the duration. This is shown in cereals when the rate of assimilate supply limits grain filling: the rate of grain development and time when maturity occurs are not greatly altered, but small grains are formed (“shrunken grain”) due to inadequate assimilate supply (Dupont and Altenbach, 2003). Similarly with other organs, if the rate of supply from the “source” is less than the demand from the young, growing “sink” then small organs with generally inferior characteristics are produced. Conversely, if the assimilate supply exceeds the demand then the genetic potential will be exceeded and other processes, such as growth of organs (e.g., late tillers) which do not contribute to yield or respiratory processes which “burn-off” the excess, may consume assimilate. This is an inefficient use of the resources consumed in production of the supply relative to the desired output.

There is not a simple link between the rate of CO_2_ assimilation (photosynthetic rate), assimilate (predominantly sucrose) production, and the dry matter of the crop and final harvested grain yield (Reynolds et al., 1996, 2011). In part this is because factors governing the production and consumption of assimilates differ qualitatively and quantitatively. Total photosynthesis per plant and of the crop is extremely dependent on the LA: photosynthetic rate per unit LA is relatively constant but LA may vary greatly. Photosynthesis and respiration are affected by environmental conditions to a different extent. Changes in light have a much greater effect on photosynthesis than changes in temperature, whereas dark respiration is not directly affected by light, but its rate is very dependent on temperature. Also, there are many processes, particularly respiration, in different parts of the plant which use assimilates required for formation of yield. They consume a varying proportion of assimilates depending on conditions. Thus, the production and consumption of assimilates are very dynamic, responding to short-term environmental conditions and metabolic demand and also over the longer-term, so the balance between source and sink must also be fast and precise but also adjusted over the long-term. **Figure 1** is a simple model of the basic plant (wheat) system with the main components and fluxes of assimilates. The sink and source are connected by the transport pathway of the phloem. Of the many materials transported, the most important are amino acids and sucrose. Phloem loading and unloading in source and sink respectively are energy-dependent, complex processes with mechanism which are unclear (Patrick and Oﬄer, 2001). Understanding of the processes involved in balancing the metabolite fluxes between “sink” and “source” is also limited, both qualitatively and quantitatively. Regulation of such a complex plant/crop system, over time has been extensively explored and there is a great body of knowledge in biochemistry (principally about the genome and metabolome), plant physiology (photosynthesis, organ growth) and crop physiology (crop development), and agronomy (yield). Although these processes are understood in physiological terms the detailed genetic and biochemical mechanisms responsible for them are not well quantified. Currently, attempts to overcome this deficiency and to improve crop production are being intensified (Foulkes et al., 2011; Reynolds et al., 2011).

All the processes are, under field conditions, subject to very rapid and widely varying environmental conditions which must be considered. It is obvious that a major determinant of yield is the supply of assimilates, both carbon and nitrogen, to the developing and growing grain yet how is this regulated in the short term when environmental conditions, such as light, cause supply to fluctuate and temperature affects demand by the sink? In addition, how is the balance in the source–sink system achieved in the long term? Specifically, what determines the relative size and functions of the sink and source organs? In principal this must be the product of genetic selection integrating all the processes contributing to both and implies a very tightly regulated mechanism. In a successful variety of a crop (i.e., one with yield which is comparable to or better than other varieties and is stable over time) this selection will principally be based on yield, and the underlying mechanisms are generally unknown. Analysis of the relation between yield and its components in wheat populations has identified the QTL enabling these factors to be linked to the (approximate) position on the chromosomes and thus, ultimately, to the underlying functional genes and mechanisms (Richards et al., 2010). Quantitative genetics, utilizing the many available molecular markers, makes it possible to analyze the genetic basis of yield, especially when QTLs for yield and other traits are employed (Quarrie et al., 2006). Hill et al. (2013) have shown that specific regions of the wheat genome affecting agronomic traits have distinct effects on specific combinations of metabolites. This indicates the growing confluence between agronomic aspects of crop performance, selection breeding using QTL and molecular markers, and the biochemistry of the whole plant system. An alternative approach is to alter the genome specifically by genetic transformation to introduce one or more novel genes or to increase or decrease the expression of an existing gene and its product to affect metabolism. This offers a way of examining the mechanisms and of altering the output of the system. In the context of this discussion of source–sink relationships and factors regulating yield, the pertinent question is what mechanism might operate to link the supply of sucrose to the processes of growth and storage. As outlined earlier, the role of trehalose in regulating the interaction of sucrose content of cells and tissues with much wider aspects of metabolism, and thus development and growth, suggests that its role in crop production should be examined in detail (Martínez-Barajas et al., 2011).

## FACTORS DETERMINING CROP PRODUCTION

### “SOURCE” PRODUCTION OF ASSIMILATES

#### Leaf scale

Carbon assimilation and assimilate production depend on total LA and its duration (LAD) which together determine the light energy intercepted, and on the efficiency with which the energy is used for the assimilation of CO_2_ (photosynthesis) into carbohydrate (sucrose) which is stored in grain as starch. This efficiency depends on a complex chain of events – from electron transport via the photosynthetic carbon reduction cycle to the distribution of the carbohydrates generated into cell components. In current advanced agriculture, crops such as winter wheat cover the land surface for a considerable period (in the UK ca 9 months) and for much of that intercept 90% of useable radiation. Increasing radiation interception with larger LAI and LAD and altered crop architecture is not likely to be very effective. Increasing the efficiency of CO_2_ (and N) assimilation/unit LA and/unit light absorbed would increase assimilate availability. This may be achieved by improving the environment, particularly nutrition: an adequate N supply at the correct time ensures that the components of the leaf responsible for CO_2_ assimilation, e.g., light harvesting and Calvin cycle (of which ribulose bisphosphate carboxylase-oxygenase (Rubisco) is the dominant component) are produced fully. As will be discussed, when assimilate supply is large, sink (grain) capacity is the limiting factor for yield. However, it is probably that the failure of much of current practical agriculture to reach the genetic potential is due to agronomy, e.g., inadequate amount or poor timing of fertilizer application or adverse growth conditions preventing exploitation of nutrients by the plant. Genetic capacity of the sinks may have been reached and exceeded with advanced agronomy but in much of the world’s agriculture inadequate agronomy and limited photosynthesis probably are the major limitation and have set an upper limit to potential grain size.

Selection breeding has not greatly improved photosynthetic rate or the enzymatic characteristics (such as Rubisco) of the photosynthetic system. Only recently have these processes been targeted, with attempts to improve the photosynthetic mechanism and thus rate (Raines, 2011). However, selection for yield ultimately integrates all plant processes and environment interactions, so it may, probably indirectly, have selected for leaf characteristics which stimulate assimilate production. Indication of this is given by the very positive correlation of flag leaf width with vegetative biomass and its association with a QTL for grain yield/ear and grain number/ear. This suggests that the traits are causally related. Wider leaves with greater number of cells and more chlorophyll per unit area are possibly related to variation in cell division during leaf ontogeny. Perhaps the gene(s) regulating yield also affect early leaf development (Quarrie et al., 2006). Assimilate may be produced in excess at times when it is not all directly used to make organs or fill grain, for example during ear and grain development when demand for sucrose is limited and the excess is then stored as fructan in stems and later remobilized, if required, to buffer assimilate supply during periods of shortage. Fructan synthesis is controlled by a sucrose-specific pathway which is perhaps affected by the transcription factor TaMYB13 (see van den Ende, 2013 for a review of this topic): it might be speculated that this system is a target for T6P regulation. Additional flexibility in the production and supply of assimilate is likely: CO_2_ assimilation may be stimulated by active sink demand, suggesting that if the sink demand could be increased then sucrose production would also rise and be used for grain filling and so increase yields. However, there would be environmental limitations to this. It is possible that current crops have been selected for particular combinations and ranges of conditions, with long-term stability of yield of great importance.

#### Plant and crop scale

Assimilate production by the plant over the period for which LA is maintained, i.e., the leaf area duration (LAD) is (grossly simplified):

Net C assimilate production = LAD × light energy intercepted/unit LA × C assimilation/unit light energy intercepted – crop respiration

LAD, both of the whole crop and particularly of the flag leaf in wheat, is a major determinant of yield, with delayed senescence of the crop canopy, and of the flag leaf specifically, positively correlated with yield. QTL analysis indicates a complex mechanism involving transfer of assimilates from source (or storage) to sink which is linked to senescence and the duration of grain filling (Verma et al., 2004).

Net C production/unit of ground surface area must be considered as it is the relevant unit of production considered in agriculture (ultimately yield is mass of product per unit land surface area):

Net C production/unit of ground surface area = Net C assimilate production/plant × plants/unit ground surface area. The importance of LA has been long-emphasized in crop physiology but is less appreciated in other disciplines.

### ASSIMILATE DISTRIBUTION TO SINKS

**FIGURE 2:**
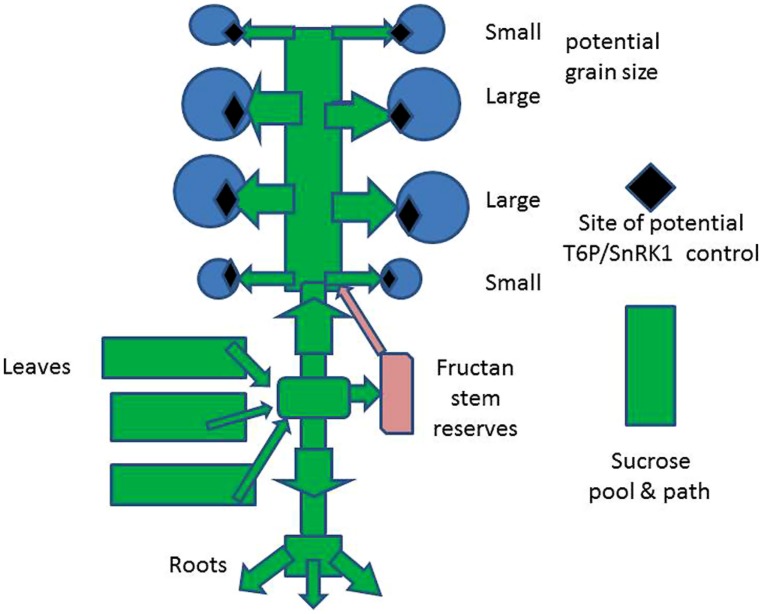
**Schematic of the source–sink relations of a wheat plant and the potential sites for modification of TRE/SnRK1 metabolism to increase potential grain size**.

Net C assimilate production is principally as sucrose which is transported throughout the plant and is partitioned into the plant’s organs: in the vegetative state sucrose supplies the requirements for structural components (e.g., cellulose in cell walls) for growth of leaves, stems, and roots and then, with the onset of the development of reproductive and storage organs, for the growth of ears and grains. Sucrose is also transported, in cereals, to stem parenchyma where it is converted to fructan (Gupta et al., 2011), which is stored (**Figure 2**): this may be remobilized and contribute to grain filling if the more direct assimilate supply from the leaves is inadequate (Latiri et al., 2013). In the reproductive stage, the endosperm of grains which have been formed is filled with starch derived from sucrose or to a smaller extent from fructan. A very important but smaller proportion of storage is protein, which is not further considered.

Patrick and Oﬄer (2001) reviewed the structures and mechanisms responsible for supply of nutrients to seeds of legumes and cereals: the following is based on their analysis. Developing seeds of cereals import organic (sucrose and amino acids predominantly) and inorganic nutrients from the phloem of the maternal vascular system where they are at relatively large concentration. However, developing seeds are isolated from the parent, in as much as there is no symplasmic linkage between the parent plant (maternal tissue) and seed (filial tissue). Transfer of nutrients from the sieve elements of the maternal phloem (located in the case of wheat in the seed crease) to the embryo and endosperm of the seed involves a complex transport system at the interface between the maternal and filial tissues. Maternal cells located at the interface have extensive plasma membranes with a large proportion of transport proteins. At the filial side of the interface are cells, in one or two layers, with membranes also enriched in transport proteins. These deliver sucrose, etc., to a highly developed system of plasmodesmata which transfer the nutrients (probably by diffusion) in the symplasm to the underlying storage cells and to the embryonic tissue and endosperm. In wheat grains encrustations in sub-aleurone cells adjacent to the starchy endosperm may indicate the location of many, very active transport mechanisms. It is, therefore, a major site for potential regulation of nutrient uptake from the adjoining apoplastic endosperm cavity, derived from tissue breakdown. Nutrients are deposited into this specialized structure which lies below the crease of the grain, the pigment strand and the nucellus (with specialized nucellar projection cells containing many mitochondria and extensive rough endoplasmic reticulum) responsible for release of nutrients from the maternal tissue into the filial tissue. The endosperm cavity lies outside the aleurone layer of the endosperm. But these structures do not constitute a large storage pool for sucrose in wheat. The transport and storage mechanisms for sucrose and proteins (which constitute the sink) and the fluxes of nutrients (which determine the growth rate of the grain) are extremely well-integrated and subject to very tight regulation with great stability in the concentrations of intermediates in the compartments. It is likely that the maternal tissue exerts considerable control over the development of the seed. (Patrick and Oﬄer, 2001). Symplastic unloading from the crease sieve elements is a primary control point (Fischer, 1996), but details of the mechanisms and their regulation are limited, although probably of the greatest importance if manipulations of such closely controlled processes are to be successful. The rate of grain filling is so very tightly regulated (see Slafer, 2010) that when assimilates coming directly from photosynthesis are limiting (as with severe water deficiency) there is remobilization of stored materials, enabling the initial rate of grainfilling to proceed at a very similar rate irrespective of conditions. However, if there is a general shortage of assimilates then the duration of filling is limited, and small and often shrunken grain result. What regulates the flux of sucrose from the phloem to the storage sites in the seed embryo and particularly endosperm is poorly understood. It seems unlikely that the phloem constitutes a limitation to grain filling. Indeed, it is clear that under conditions which are conducive to photosynthesis the seed itself regulates the flux in relation to its growth rate. This results in seed-limited production of grain and of crop yield as discussed later.

Understanding regulation of such a structurally and functionally complex system presents considerable difficulty, and it is unclear how it could be manipulated, e.g., by altering T6P metabolism. However, analysis of a manipulated grain system would, with some certainty, provide insight into the processes.

### YIELD COMPONENTS

As in equations above, the crop components determining net C accumulation and ultimately harvested yield may be identified (Slafer and Araus, 2007). For a cereal plant:

Net C yield/plant = number of tillers × number of ears/tiller × number of grains/ear × mass C/grain

And per unit land area:

Net C yield/unit ground area = Net C yield/plant × number of plants/unit land area.

The net C yield/unit ground area is the harvested yield of carbon and this may be converted to the total crop yield, given the proportion of C to total mass harvest mass. At harvest the ratio of the dry masses of the yield to total crop (vegetative organs plus the yield) is the harvest index (HI).

Clearly, the number of “structures” contributing to yield of a crop is considerable, and production of yield involves not just photosynthesis but many growth processes. Focusing on either assimilate production or growth does not provide a sufficient framework for understanding the formation of crop yield (Reynolds et al., 1996, 2011). Consequently, the interactions between processes should also be addressed. To improve yields, relevant structures must be increased and this will require that the processes leading to their formation must be adjusted. For example, to increase grain yield it is theoretically possible to increase grain number (the predominant method historically by selection breeding; Slafer, 2010) via the number of plants/unit land area, or the number of ears/tillers or tillers/plant, or the number of grains/ear. Indeed, in agronomic terms the option of altering the number of plants/area by changing the number of seed sown is of considerable importance. The success of this will depend on seed viability under the prevailing conditions and on the later conditions experienced by the crop. There will also be interactions between the number of plants and the LA and crop architecture which may affect crop yield. Here it is important to highlight the uncertainty in many agronomic activities: the choice made by farmers is based on the long-term success of a particular action. In specific growing seasons the number of seed/area may be suited to the conditions and the yield is excellent but with other conditions the result is poor production. Altering a plant’s genotype in a particular way may not achieve the desired effect when the plant is grown as a crop in a variable environment. Grain number is the most important variable component of yield and mass/grain the most conservative. The increase in grain mass starts shortly after anthesis and is very constant under a wide range of conditions (Frederick and Bauer, 1999; Slafer et al., 2009) and proceeds until the potential size is reached or assimilate supply is inadequate (under severe assimilate deficiency). This constancy suggest that the process of obtaining assimilates from the parent plant is genetically determined and strongly regulated (Fischer and Edmeades, 2010).

### YIELD LIMITATIONS: GRAIN NUMBER AND SIZE

Extensive and detailed analyses of yield and its dependence on grain number, size, and assimilate supply have been made for wheat from many sources and has been summarized by Miralles and Slafer (2007), Slafer et al. (2009), Slafer (2010), and Distelfeld et al. (2014) regarding sink and source limitation in crops of widely different characteristics. As already mentioned, there is the generally observed positive relationship between the total yield/ha and grain number/ha, made up of number of ears/ha, and the number of grains/ear. This is independent of the age of the variety (i.e., when first introduced) or conditions, e.g., timing and amount of nitrogen fertilizer supplied. However, the number of grains is inversely related to the mass/grain. This has generally been interpreted as the effect of competition for assimilates between grains. In consequence it is usually considered that the supply of assimilates is limiting crop yield, and the corollary is that if photosynthesis could be increased so the yield would increase. The view has become established that crops of wheat are source – not sink – limited. However, manipulating the source–sink balance by defoliation or de-graining wheat plants provides conclusive evidence that it is the sink which is limiting (Slafer, 2007). Even in Mediterranean environments with a 100% difference in assimilates available per grain, grain mass of old and modern varieties of bread and durum wheat only increased by a maximum of 15% (Miralles and Slafer, 2007; Acreche et al., 2009). This suggests that competition between grains is limited and that, even when assimilate is abundant, mass/grain is conservative. An increase in crop yield proportional to grain number will not occur, because the sink is limited, not because of source limitation. Grain number has been the predominant feature selected for in breeding wheat. There is an inverse relation between grain number and mass/grain (Slafer et al., 2009; Cossani et al., 2010; Slafer, 2010) despite the apparent constancy of grain mass. What causes the inverse relation between mass/grain and number of grain if there is little or no competition for assimilates during grain filling? The answer is that the increase in grain number is associated with a decrease in the average potential grain size. Grain at the extremes of the ear and also on lower-ranking tillers have grain of smaller genetic potential capacity to accumulate assimilates. With an adequate assimilate supply the grains will fill but as the potential size is limited the grains are small, hence the negative relation between grain number and average mass. It has been suggested that this formation of more small grains when the capacity to make large grains is satisfied had advantages in evolutionary terms, as large, fully filled grain will have greater “fitness” than smaller grains. Altering grain number via tiller production, which is sensitive to environmental conditions, e.g., water deficits, is a flexible adaptive mechanism. Distributing limited assimilate across a large number of grain would decrease fitness. However, if assimilate is available then smaller grain will fill and may later germinate and reproduce, so taking advantage of good conditions to increase the population of plants in an area. In this context, when assimilate supply from leaves is very restricted, e.g., with severe water deficits, grains may only partly fill and others abort as there is inadequate assimilate to fill all the grains established during development, despite stored fructans (**Figure 2**). Grains with small potential at the base and tip of the ear do not acquire assimilates under these conditions and are very small (shrunken) or they abort. As discussed, this regulation of grain size is important in determining average grain mass in the whole yield but also ensures that viability of the next generation of plants is maintained (grain reserves are essential for early seedling vigor – growth). Slafer (2010) and Foulkes et al. (2011) conclude that it is important to increase the potential grain size to increase the average mass/grain and thus increase yield and that this would function with the CO_2_ assimilation capacity of current wheat varieties.

### INCREASE THE NUMBER AND/OR CAPACITY OF STORAGE ORGANS (“SINK”)

Grain yield can be increased by increasing either total biomass, which means more and larger source and sink organs, or the HI which requires more grains or larger grains (although all these may contribute – they are not mutually exclusive; Quarrie et al., 2006). By altering growth of the meristems of tillers, ears, and grains a larger crop sink could be achieved (Slafer and Araus, 2007). As described earlier this could be by different routes. Selection breeding, using molecular methods to identify the most direct routes to change the plant, indicates the trends in selecting for superior yields in wheat. Selection for grain number has certainly been a major method – as shown by the great range of combinations of ear number and grains/ear seen in current high-yielding cultivars of wheat. There is evidence that increasing grains/per ear, rather than increasing the number of ear-bearing tillers, has been important, with increasing grain mass playing a relatively small part (Gegas et al., 2010). Thus it seems that increases in yield might result from alterations to the genome which ultimately increases these components. In durum wheat, as in winter wheat and other cereals, there are a few major QTL for yield with relatively large effect, acting together with many minor QTL. They are greatly influenced by environmental conditions. Comparison of QTL for different traits in a wide range of genetic material shows that they are generally clustered. This is common for traits that affect plant growth, the responses to environmental conditions and yield (Maccaferri et al., 2008). However, although the location of the traits on chromosomes is known, the nature of the genes responsible is not. The yield component most strongly associated with these QTL was grains per ear, with a close link to biomass (Quarrie et al., 2006): plants growing at, or close to, the genetic potential have (by definition) large assimilate production and thus form many grains and fill them fully. These yield traits are also associated with a wide range of leaf numbers and sizes (area per leaf, leaf blade width, plus angles, and orientations). Thus, increasing yield will require further detailed analysis of the mechanisms which are responsible for both assimilate production and its storage. Both are potentially determined by the development of the cells, both structure and composition.

There is no evidence from breeding and genetic analysis that T6P metabolism is related to the QTLs identified. However, a gene related to branching inflorescence branching in maize (*RAMOSA3*) has been identified as a trehalose-6-phosphate phosphatase by Satoh-Nagasawa et al. (2006), who proposed that *RAMOSA3* regulated the branching of the inflorescence by altering signals associated with sucrose. It is unclear if the product of this gene is an active T6P phosphatase (Satoh-Nagasawa et al., 2006; O’Hara et al., 2013). Possibly by examining the role of T6P in signaling the sucrose status of the plant during grain growth and filling and linking it, genetically, to effects on the plant and yield better understanding of the putative role of T6P/SnRK1 might be obtained. Current evidence is that T6P does alter many processes in cell development and growth, plus altering the source/sink relations (perhaps as a consequence of changing structural features). Refinement of such changes may have the potential to improve yield. Evidence that the morphological and functional changes associated with increasing yield have been achieved during selection and breeding by specific metabolic processes and are associated with genomic changes is lacking. Ultimately, the combination of transcriptomics, proteomics, metabolomics, and QTL analysis has the potential to reveal interactions and relationships among genes, transcripts, proteins, metabolites, and traits (Hill et al., 2013). This is a huge task and direct modification of the system with specific changes, as with T6P transgenics, and their analysis offers a way of showing what processes may be effectively targeted.

An indication of the potential mechanisms which might be altered and of the effects on the plant and crop is provided by the transformations already made to the T6P/SnRK1 pathway. When combined with knowledge of how this regulatory system functions it provides a way of potentially increasing yields and of minimizing dependence on environmental factors. Exploiting the mode of action of T6P is already advanced by genetic modification. It may be assumed that there is a T6P/SnRK1 system in place in current, non-transformed plants that is genetically “set” and has been selected during breeding. If sucrose is abundant, T6P is also in large concentration and metabolism and growth are stimulated. If the sucrose supply is limited then the T6P concentration would also be small so allowing SnRK1 to phosphorylate target proteins. This provides a mechanism for balancing source and sink. The question might be posed: is it possible to reset this constitutive balance by altering expression of trehalose pathway genes? Constitutive over-expression of T6P causes a number of changes to growth and function due to imbalanced metabolism throughout the plant, as metabolic processes compete. By directing expression of T6P to specific organs it is likely that more effective increase in sink capacity will be achieved. Increased T6P would inhibit SnRK1 giving active target proteins (and also regulate other protein kinases) to increase growth or capacity of sinks – ear and grain. This would simulate an increased sucrose concentration and enhanced availability of sucrose. Of course, the source–sink balance might be altered: if sucrose from the source is limiting then growth, etc., would not take place, or the transformed organ might act as a dominant sink and use the sucrose supply so depriving another organ. This increased in-plant competition might not impair yield if assimilate supply is large but could do so if supply is inadequate. The effects would require evaluation.

### ALTERING MASS/GRAIN

**FIGURE 3:**
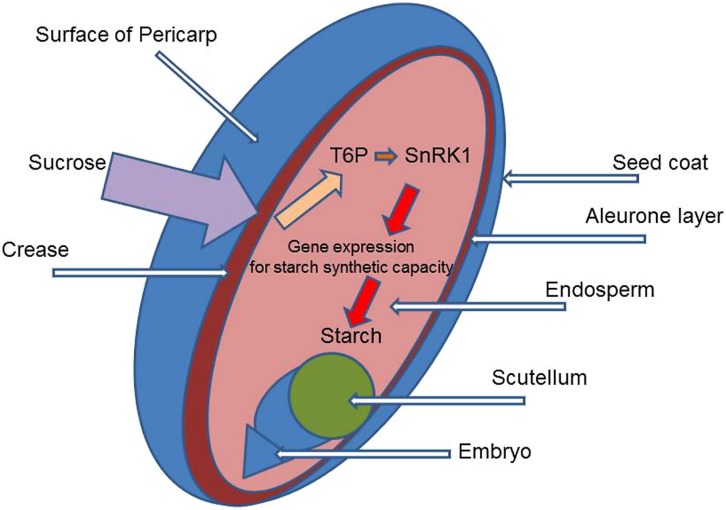
**Diagram of a wheat grain identifying the main structural components and site for potentially increasing grain size by altering TRE/SnRK1 metabolism**.

Increasing the potential size of grain would probably require intervention in the meristems of ears and grains at the early stages of their differentiation from the vegetative apex into reproductive structures, even before “terminal spikelet” formation. However, it could also occur in the very early period of grain development, after anthesis. This would involve increasing cell division and cell growth to give larger cells, with greater potential for assimilate storage. It would seem likely that the capacity of the endosperm, the number and size of the cells formed and the content of the enzyme systems for the conversion of sucrose into starch and for making starch grains, is the key determinant of yield, for it is there that starch synthesis and deposition occur (**Figure 3**). (Storage proteins – gliadins and glutenins – are also synthesized and stored and is coordinated with starch metabolism: for a detailed review of metabolism in grain see Dupont and Altenbach, 2003). Development of the endosperm (a triploid tissue) soon after fertilization may influence final grain size in wheat as in maize (Engelen-Eigles et al., 2000). These processes are very sensitive to environmental conditions which influence grain number and potential size. Clearly events during this period would be a potential target for modification to increase yield and alter grain quality. In relation to T6p/SnRK1 metabolism, specific over-expression of TPS/TPP in meristems giving ears and grains with increased T6P content would be one method to stimulate cell division and growth of the cells so increasing potential grain size. Another possibility is to enhance gene expression to stimulate the synthesis of components of the sucrose transport system and also enzymes for conversion of sucrose to starch in the endosperm (Emes et al., 2003). Increasing T6P in specific tissues and at critical periods in development could have the effect of increasing sink strength, as deposition of starch would decrease the concentration of sucrose allowing greater flux to the sink. The complexity of starch synthesis in the endosperm should be mentioned with synthesis of ADP-glucose from glucose 1-phosphate by ADP-glucose pyrophosphorylase. ADP-glucose is converted to the polymers of amylase and amylopectin by starch synthases and branching enzymes located exclusively within plastids and deposited in large type-A granules initially (4–7 days after anthesis and then later (10–12 days after anthesis) in smaller type-B granules (Emes et al., 2003). T6P clearly plays a role in starch synthesis (Martínez-Barajas et al., 2011; Nunes et al., 2013b) but in which part of the mechanism is not known. The number of ears and grains formed, and the capacity of endosperm cells to make store starch grains are clearly of primary importance but they would probably not act alone: rather they would interact strongly with phytohormones (Lisso et al., 2013) which are responsible for relating organ development to other processes in the plant and to environmental conditions such as light. Plants with modified trehalose metabolism would require testing in target environments, with the natural range and combination of conditions: as explained earlier a strong GXE interaction might be expected.

### TIMING OF METABOLIC EVENTS IN GRAIN DEVELOPMENT AND FILLING

The number of grain/ear is positively related to the mass of the ear at anthesis, so a crop growing under good conditions will form bigger ears, as well as more ears/plant and per unit area and also grains with greater potential capacity for starch (and protein) storage. With poor conditions fewer ears, with fewer and potentially smaller grains, will be formed. Conditions 10–15 days before, and up to 10 days after anthesis, are very important as the very early stages of ear and grain establishment and growth are very sensitivity and establish the number of grains and the grain characteristics respectively. The rapid growth of the ear results in substantial demand for assimilates. Hence, if assimilate supply is good then a larger number of grains is formed than if they are poor. However, within the developing ear a hierarchy of “sink-strength” is established so under all situations some grain with small potential and final size (mass) are formed. The duration of the period in which floret/ear characteristics is formed is very brief, so the rate of supply of assimilate to cells is probably at its maximum then, hence the sensitivity to conditions. Also, it seems that breeding and selection have (Slafer, 2010) focused, subconsciously, on this period and processes maximizing potential grain number. Changing the assimilate supply experimentally by altering the radiation experienced by crops by shading and growing at different CO_2_ concentration before and particularly at anthesis, showed how critical conditions affecting assimilates supply is for establishing the number of fertile florets or grains and also for the potential size of grain ultimately established in the ear (Mitchell et al., 1996). However, there are complex interactions between conditions and effects on potential grain size. It is unclear what mechanisms are operating at this time (Dupont and Altenbach, 2003) but there are relationships with crop biomass suggesting that availability of sucrose is crucial. In the early growth stages the florets within the ear and the grain are developing rapidly. Cell division and expansion require substrates for construction of cell components and as energy sources, and the correct conditions (temperature, for example). At about the same time as stem elongation is occurring and at anthesis there may be competition for assimilates – sucrose and amino acids – between ear/floret development and growth and stem growth. Also fructan reserves may be accumulating. Perhaps this stage was crucial under natural conditions and in evolutionary, as it is now in agricultural, terms: poor growing conditions act as a signal that the conditions during the next period of ca 30–40 days during which the grain fill have a large probability of also being poor or even worse (drought for example). At this stage, and for only a brief period, the developing the young meristems must receive the required materials to establish the potential size of the grain. At this time positional effects occur which determine potential grain size within the ear and on tillers. This is probably genetically determined as it occurs irrespective of assimilate supply. Growth of wheat plants in elevated CO_2_ (see Mitchell et al., 1993, 1996) indicates the importance of assimilate supply at particular times for grain components. Increased sink capacity would only increase yield if sufficient assimilate were available at the critical time, to establish many grains of large potential size, and then, post anthesis, to fill the grain for the period necessary to achieve the potential. There are conditions (severe drought for example) which may decrease assimilation substantially so that potential size is not attained. However, in well fertilized and watered crops the assimilate supply should enable the larger capacity to be established. The problem may be seen as one of optimization of source and sink, and of the timing of events, against a background of a variable environment and agronomic practices (Foulkes et al., 2011). The biochemical mechanisms responsible are critical to understanding what determines agricultural yield (Lawlor, 2002a). It will always be the case that the match between potential of the plant, determined by the genome, and the environment will always be fluctuating around a long-term optimum. However, the mechanisms by which source and sink are balanced in the crop, over a long period, and under varying environmental conditions are very poorly understood. How to increase grain size (and rate of filling) is unclear. Given its conservative nature and perhaps limited genetic variability, direct intervention by genetic manipulation, for example by altering trehalose metabolism may provide a method and certainly would increase understanding of regulation of grain development and growth.

## POTENTIAL ROLE OF T6P/SnRK1 IN CELL METABOLISM

From the above analysis of yield and its components it is immediately apparent that the yield of a crop depends on many factors which determine the number and capacity of the organs to make and store assimilates, plus those that determine the transport and deposition of storage materials into the harvested organs. The number of stems, leaves, ears, and grains is determined during vegetative growth and the early reproductive phase by development of meristems. The size of all organs depends on the number of cells in the organ and the size of the cells, and the structure of organs depends on the relative proportion of cell types constituting the tissue and the way in which they are distributed. The rate of production of organs depends on the rate of cell division in the meristems and the rate of cell growth (expansion) as cells exit from the zone of division. The maximum possible size of cells is, to a first approximation, determined by the genetic potential that is the activity of all the gene-based processes which form the cell. Defining the genetic processes is difficult but progressing. This potential can only be achieved when the supply of assimilates and the environmental conditions are not limiting and optimal, respectively, at crucial times in development and growth. There is considerable uncertainty about what mechanisms are responsible for regulating development and growth in relation to environment and assimilate production.

We hypothesis, on the basis of the evidence, that T6P/SnRK1 is the mechanism by which the relative size and metabolic activity of cells in source and sink organs are determined. It operates together with other mechanisms such as those determining the supply of amino acids and the synthesis of proteins, to regulate the cell division and synthesis of components in relation to sucrose supply. Further, growth and function of cells determining potential grain size may be altered by manipulating the synthesis and breakdown of T6P. Large sucrose concentrations, via T6P and its regulation of SnRK1 would stimulate meristem cell growth and division and cell expansion, and perhaps give cells, and ultimately organs (grains) with a large capacity for sucrose transport, with many storage sites and a large activity of enzymes, etc., for later starch accumulation. The T6P/SnRK1 mechanism would detect the concentration of sucrose in the supply pool to the organ and thereby increasing gene expression and protein synthesis, so altering the activity of transporters or other components (enzymes responsible for starch synthesis). This would be a “high potential” grain. Conversely limited sucrose synthesis and a small sucrose concentration would result in a grain with much decreased capacity and “low potential.” The mechanism might also function in altering the balance of processes under other conditions. In the plant, manipulation of T6P metabolism in different organs may regulate the relative fluxes to them, for example improving the rates of filling of grains relative to vegetative organs. Such a role of T6P is indicated by the effects of increasing T6P by transformation of plants. Increasing expression selectively, in ears for example, would alter SnRK1 and the capacity for sucrose accumulation and possibly also sucrose allocation.

The model based on T6P as a sensor of sucrose status, suggests that if the sucrose content decreases, as with limited CO_2_ supply, then the fall in T6P leads to activation of SnRK1, and metabolic reprogramming so decreasing synthesis of particular proteins related to cell division and cell wall formation. The evidence from such studies is that division of cells is decreased and growth of cell walls is also affected (Gómez et al., 2006). Expansion growth of cells would be decreased as their walls no longer expand as rapidly or fully as in elevated CO_2_ or when sucrose is more available. Consequently, cells are smaller giving smaller organs: LA decreases so decreasing light interception and reducing the competitive advantage. However, the content of cell structures responsible for metabolism, such as mitochondria and chloroplasts, may not be decreased in proportion to the smaller size (volume) of the cells. The result is leaves which have a relatively large content/unit area of metabolic machinery and so are relatively more efficient whereas decreased expression leads to expansion of cells and decreases photosynthesis rates (see Pellny et al., 2004). More efficient photosynthesis would maintain the sucrose content and so stimulate growth by inhibiting the SnRK1 and preventing the inhibition of anabolic processes. The mechanism would link the photosynthetic production of carbohydrates, specifically sucrose, which is transported throughout the plant, to growth of cells and organs. The relative effects on the structure of the plant – in terms of proportions of organs and storage components and storage material – will depend on a number of factors and processes: the severity of the N deficiency relative to CO_2_ supply, rates of photosynthesis and respiration, and the stage of the plant’s development.

### POSSIBLE ROLE OF T6P/SnRK1 IN WATER DEFICIENT CELLS AND PLANTS

Considerable effort has been made to breed wheat varieties which are more productive than existing ones under water deficits (Richards et al., 2010) with some success, mainly under mild and fluctuating water supply. However, many forms of genetic modification of plants have resulted in claims to have achieved substantial drought resistance which would be beneficial under more extreme droughts and plant water deficits. The form of resistance obtained delayed stress onset (Lawlor, 2013) – is from slowing the rate of water loss either by decreasing the leaf are of the plant or decreasing the stomatal conductance, or a combination of the two. Owing to inadequate concepts of what drought resistance means and unsatisfactory methods of testing for the potential mechanisms, there is no definitive evidence that modifications have induced metabolic changes which have increased inherent drought resistance (tolerance) as opposed to altering leaf growth and area and stomatal behavior which determine the rate of water loss (see Lawlor, 2013 for a full discussion). Modifying TRE metabolism and content has been proposed to stimulate growth substantially not only under good environmental conditions, but also when these are inadequate, particularly under drought. Holmström et al. (1996) showed delayed stress onset in tobacco plants transformed with TPS1 caused by slower transpiration resulting from smaller plants and decreased stomatal conductance rather than a demonstrated metabolic form of drought tolerance. Expression of the TPS from yeast in potato plants also slowed water loss from leaves and delayed stress onset by decreasing stomatal density and therefore stomatal conductance and slowing growth of LA, which was probably smaller than the wild-type plants (Stiller et al., 2008). Similarly, transformation of tobacco chloroplasts with yeast TPS1 gene decreased growth rate of To plants but not later generations compared to the wild-type and was considered to confer drought tolerance (Lee et al., 2003). Plants transformed to increase the content of T6P and TRE were more “drought resistant” than the wild-type (Han et al., 2005) but this was stated not to affect growth. Similarly, Karim et al. (2007) used constructs which did not to affect growth but did slow water loss. It is now generally recognized (Fernandez et al., 2010) that modifying TRE metabolism affects plant growth and leaf structure, with disturbance of stomatal development, affecting the stomatal apparatus, altering the number of stomata per unit area (density) or their function, decreasing the stomatal conductance to water vapor and so slowing water loss and apparently giving drought tolerance. Increased carbohydrate accumulation might contribute to osmotic adjustment but TRE itself does not, nor does it increase cell water retention by decreasing the osmolyte content. The potential for modifying TRE/T6P metabolism to improve crop growth and yields under drought conditions has been frequently addressed. In a recent review Delorge et al. (2014) did not differentiate between the “drought resistance” caused by smaller LA and decreased stomatal conductance slowing the rate of water loss and thus the rate of stress onset, from intrinsic metabolic resistance. They emphasize the possibility of modifying trehalose metabolism by over-expression of trehalase to alter stomatal behavior, specifically closing stomata during drought. From the analysis they develop an “optimal plant” although the complex interactions known to occur between decreasing stomatal conductance, water loss by the plant and photosynthesis are not considered although it is recognized that “tight regulation of stomatal movements is very important during drought stress as this regulates optimal water and CO_2_ exchange.”

Given the information it is reasonable to postulate the following mechanism in leaf tissues and cells of young, growing plants when they are exposed to relatively slowly developing, mild water deficit. Photosynthesis is less inhibited than expansion growth of cells and organs. So sucrose concentration rises, which increases T6P and inhibits SnRK1, maintaining or increasing synthesis of cell walls and cell division. It is well-known that cell division is maintained under drought but only when water becomes available do cells expand, allowing leaves to grow very rapidly for a period. This is a possible mechanism allowing adjustment of growth to the water supply. However, if the water deficit is more severe with smaller relative water content and stomatal conductance so that CO_2_ assimilation decreases more than demand, sucrose synthesis, and concentration fall, leading to a smaller concentration of T6P. This increases SnRK1 activity. The consequent phosphorylation of target proteins inhibits many aspects of metabolism, for example NR and SPS activity. Also inhibition of protein synthesis is likely. Increasing the sucrose supply under these conditions would overcome the inhibition of NR and SPS if the main effect of water deficit was on stomatal conductance and thus on CO_2_ supply. Hence, any effect of increasing T6P would simulate an increase in sucrose synthesis, even if this were not the case. This would explain why elevated CO_2_ reversed the effect of water deficit on NR activity (Kaiser and Förster, 1989). However, water deficit of this type also results in inhibition of ATP synthesis (Lawlor, 2002b). As discussed the adenylate kinase system results in a large AMP/ATP ratio which activates SnRK1 and would inhibit activities of their target enzymes. Thus, the effects of water deficit would be very dependent on its severity, and on the relative effects of changes in T6P and ATP. Increasing, by transformation, the T6P content to inhibit the SnRK1 activity and so stimulate metabolic activities, e.g., synthesis of cellular proteins and metabolites, might have a protective role under drought. Such a mechanism would explain the phenomenon of “hardening” which is a feature of plant adjustment to environmental conditions, e.g., cool and low temperature, salinity, etc. However, increased T6P content, simulating a large sucrose content, might conflict with the need to inhibit synthetic processes and conserve resources to maintain basic metabolic processes required for cell, tissue and organ survival under adverse conditions. If T6P were increased in organs such as the ear or specific grains so stimulating or maintaining the movement of assimilates (either directly from photosynthesis of leaves or from storage such as fructans in stems) to them at a time of general sucrose shortage this might be beneficial, allowing grain growth to continue. A consequence would be competition with, say vegetative tissues or with organs of smaller sink-strength. The attraction of such modification to T6P metabolism is that it would provide the plant and crop with a very flexible and highly-tuned regulatory mechanism to deal with fluctuations in light and CO_2_ (via stomatal conductance and water supply) which affect the sucrose pool, but with a greater ability to form and fill grains under adverse conditions than non-modified plants. Critical experimentation under a range of conditions would be required to test such an hypothesis.

### POTENTIAL AND KNOWN RESULTS OF MODIFYING TREHALOSE METABOLISM IN PLANT

The idea of modifying grain yield of wheat by altering T6P content of specific tissues to increase the potential grain size is appealing. However, it should set against the evidence from many studies which shows that transformed plants do not grow and yield in expected, or hoped for, ways. Transformed potato (Debast et al., 2011) grew poorly compared with the untransformed, producing a small yield of many small tubers, and the dormancy was affected. Indeed, the effects of altered trehalose metabolism was initially obvious from the effects on growth and metabolism. In this trehalose is not unique: transformations of many types affect plant size and stomatal function, generally impairing them. This is the cause of the apparent “drought resistance” so often claimed (Lawlor, 2013). Large changes to metabolism of systems which are very highly integrated and optimized systems are likely to have negative effects (Primavesi et al., 2011; Lunn et al., 2014). There is evidence that some types of genetic modifications may have positive effects, for example photosynthetic rate and (to a smaller extent) dry matter production can be increased by modifying the enzymes of the Calvin cycle (Raines, 2011). Transformation to alter T6P in wheat and wheat grain would provide a way of testing the practical feasibility of some of the ideas discussed. Also, it would provide an opportunity to examine what biochemical factors determine the development of grain and their potential and actual growth. Considering such effects in the context of metabolic modeling has the potential to advance understanding (Williams et al., 2010). This might help to “break–open” the black box of regulation of grain potential size for which there is limited understanding. Advancing analysis of what determines key steps in production of wheat, one of the major crops, is a major challenge.

## CONCLUSION

The importance of trehalose metabolism, and specifically the content of T6P as a sensor of sucrose concentration and availability in plants, is established. T6P modulates the activity of protein kinase SnRK1 and this has large consequences for cell division, development, and function so determining growth and yield of plants. Genetic modification of T6P has substantial effects on plant development, growth, and photosynthesis so offering a way of changing crops. The growth and harvested yield of crops depends on basic cellular processes which determine the number, size, and storage capacity of the relevant organs. The components determining crop grain yield in wheat are grain number, determined by ear production and grains per ear, and mass/grain. It is desirable to increase mass/grain to increase the sink capacity which is limiting yield rather than photosynthetic capacity. Altering T6P/SnRK1 in meristems and young grains offers a mechanism to potentially increase potential grain size to improve yields. However, how the T6P/SnRK1 system functions in crop production and how it may be exploited to increase yield and efficiency are not well explored. This review attempts to set-out possible ways that T6P/SnRK1 could operate in the context of the plant and crop factors which determine yield and makes the case for investigations which may have practical consequences and will certainly advance understanding of grain growth and crop yields.

## Conflict of Interest Statement

The authors declare that the research was conducted in the absence of any commercial or financial relationships that could be construed as a potential conflict of interest.

## References

[B1] Acreche M. Briceño-Félix G. Martín Sánchez J. A. Slafer G. A. (2009). Grain number determination in an old and modern Mediterranean wheat as affected by pre-anthesis shading. *Crop Past. Sci.* 60 271–279 10.1071/CP08236

[B2] Almeida A. M. Cardoso L. A. Santos D. M. Torné J. M. Fevereiro P. S. (2007) Trehalose and its applications in plant biotechnology. *In Vitro Cell Dev. Biol. Plant* 43 167–177 10.1007/s11627-006-9024-3

[B3] Araus J. L. Slafer G. A. Royo C. Serret M. D. (2008). Breeding for yield potential and stress adaptation in cereals. *CRC Crit. Rev. Plant Sci.* 27 377–412 10.1080/07352680802467736

[B4] Cossani M. C. Slafer G. A. Savin R. (2010). Co-limitation of nitrogen and water, and yield and resource-use efficiencies of wheat and barley. *Crop Past. Sci.* 61 844–851 10.1071/CP10018

[B5] Debast S. Nunes-Nesi A. Hajirezaei M. R. Hofmann J. Sonnewald U. Fernie A. R. (2011). Altering trehalose-6-phosphate content in transgenic potato tubers affects tuber growth and alters responsiveness to hormones during sprouting. *Plant Physiol.* 156 1754–1771 10.1104/pp.111.179903 21670224 PMC3149945

[B6] Delatte T. L. Sedijani P. Kondou Y. Matsui M. de Jong G. J. Somsen G. W. (2011). Growth arrest by trehalose-6-phosphate: an astonishing case of primary metabolite control over growth by way of the SnRK1 signaling pathway. *Plant Physiol.* 157 160–174 10.1104/pp.111.180422 21753116 PMC3165867

[B7] Delorge I. Janiak M. Carpentier S. Van Dijck P. (2014). Fine tuning of trehalose biosynthesis and hydrolysis as novel tools for the generation of abiotic stress tolerant plants. *Front. Plant Sci.* 5:147 10.3389/fpls.2014.00147 PMC3995065 24782885

[B8] Distelfeld A. Avni R. Fischer A. M. (2014). Senescence, nutrient remobilization, and yield in wheat and barley. *J. Exp. Bot.* 14 3783–3798 10.1093/jxb/ert477 24470467

[B9] Dupont F. M. Altenbach S. B. (2003). Molecular and biochemical impacts of environmental factors on wheat grain development and protein synthesis. *J. Cereal Sci.* 38 133–146 10.1016/S0733-5210(03)00030-4

[B10] Emes M. J. Bowsher C. G. Hedley C. Burrell M. M. Scrase-Field E. S. F. Tetlow I. J. (2003). Starch synthesis and carbon partitioning in developing endosperm. *J. Exp. Bot.* 54 569–575 10.1093/jxb/erg089 12508067

[B11] Engelen-Eigles G. Jones R. J. Phillips R. L. (2000). DNA endoreduplication in maize endosperm cells: the effect of exposure to short-term high temperature. *Plant Cell Environ.* 23 657–663 10.1046/j.1365-3040.2000.00564.x

[B12] Fernandez O. Béthencourt L. Quero A. Sangwan R. S. Clément C. (2010). Trehalose and plant stress responses: friend or foe? *Trends Plant Sci.* 15 409–417 10.1016/j.tplants.2010.04.004 20494608

[B13] Fischer R. A. Edmeades G. O. (2010). Breeding and cereal yield progress. *Crop Sci.* 50 85–98 10.2135/cropsci2009.10.0564

[B14] Fischer R. A. (1996). “Wheat physiology at CIMMYT and raising the yield plateau,” in *Increasing Yield Potential in Wheat: Breaking the Barriers: Proceedings of the Workshop Held in Ciudad Obregn Sonora Mexico* eds Reynolds M. P. Rajaram S. McNab A. (Mexico, DF: CIMMYT) 238

[B15] Foulkes M. J. Slafer G. A. Davies W. J. Berry P. M. Sylvester-Bradley R. Martre P. (2011). Raising yield potential of wheat. III. Optimizing partitioning to grain while maintaining lodging resistance. *J. Exp. Bot.* 62 469–486 10.1093/jxb/erq300 20952627

[B16] Frederick J. R. Bauer P. J. (1999) “Physiological and numerical components of wheat yield,” in *Wheat: Ecology and Physiology of Yield Determination* eds Satorre E. H. Slafer G. A. (Taylor and Francis) 45–65

[B17] Garg A. K. Kim J.-K. Owens T .G. Ranwala A .P. Choi Y .D. Kochian L .V. (2002). Trehalose accumulation in rice plants confers high tolerance levels to different abiotic stresses. *Proc. Natl. Acad. Sci. U.S.A.* 99 15898–15903 10.1073/pnas.252637799 12456878 PMC138536

[B18] Gegas V. C. Nazari A. Griffiths S. Simmonds J. Fish L. Orford S. (2010). A genetic framework for grain size and shape variation in wheat. *Plant Cell* 22 1046–1056 10.1105/tpc.110.074153 20363770 PMC2879751

[B19] Gómez L. D. Baud S. Gilday A. Li Y. Graham I. A. (2006). Delayed embryo development in the *ARABIDOPSIS TREHALOSE-6-PHOSPHATE SYNTHASE 1* mutant is associated with altered cell wall structure, decreased cell division and starch accumulation. *Plant J.* 46 69–84 10.1111/j.1365-313X.2006.02662.x 16553896

[B20] Guérinier T. Millan L. Crozet P. Oury C. Rey F. Valot B. (2013). Phosphorylation of p27KIP1 homologs KRP6 and 7 by SNF1-related protein kinase-1 links plant energy homeostasis and cell proliferation. *Plant J.* 75 515–525 10.1111/tpj.12218 23617622

[B21] Gupta A. K. Kaur K. Kaur N. (2011). Stem reserve mobilization and sink activity in wheat under drought conditions. *Am. J. Plant Sci.* 2 70–77 10.4236/ajps.2011.21010

[B22] Halford N. G. Hey S. J. (2009). Snf1-related protein kinases (SnRKs) act within an intricate network that links metabolic and stress signalling in plants. *Biochem. J.* 419 247–259 10.1042/BJ20082408 19309312

[B23] Han S.-E. Park S.-R. Kwon H.-B. Yi B.-Y. Lee G.-B. Byun M.-O. (2005). Genetic engineering of drought-resistant tobacco plants by introducing the trehalose phosphorylase (TP) gene from *Pleurotus sajor-caju*. *Plant Cell Tissue Organ Cult.* 82 151–158 10.1007/s11240-004-8124-1

[B24] Hill C. B. Taylor J. D. Edwards J. Mather D. Bacic A. Langridge P. (2013). Whole genome mapping of agronomic and metabolic traits to identify novel quantitative trait loci in bread wheat grown in a water-limited environment. *Plant Physiol.* 162 1266–1281 10.1104/pp.113.217851 23660834 PMC3707548

[B25] Holmström K. O. Mäntylä E. Welin B. Mandal A. Tapio Palva E. Tunnela O. E. (1996). Drought tolerance in tobacco. *Nature* 379 683–684 10.1038/379683a0

[B26] Iordachescu M. Imai R. (2008). Trehalose biosynthesis in response to abiotic stresses. *J. Integr. Plant Biol.* 50 1223–1229 10.1111/j.1744-7909.2008.00736.x 19017109

[B27] Jeong J. S. Kim Y. S. Baek K. H. Jung H. Ha S.-H. Choi Y. D. (2010). Root-specific expression of *OsNAC10* improves drought tolerance and grain yield in rice under field drought conditions. *Plant Physiol.* 153 185–197 10.1104/pp.110.154773 20335401 PMC2862432

[B28] Jonak C. Ligterink W. Hirt H. (1999). MAP kinases in plant signal transduction cell. *Mol. Life Sci.* 55 204–213 10.1007/s000180050285 PMC11147126 10188583

[B29] Kaiser W. M. Förster J. (1989). Low CO_2_ prevents nitrate reduction in leaves. *Plant Physiol.* 91 970–974 10.1104/pp.91.3.970 16667163 PMC1062103

[B30] Karim S. Aronsson H. Ericson H. Pirhonen M. Leyman B. Welin B. (2007). Improved drought tolerance without undesirable side effects in transgenic plants producing trehalose. *Plant Mol. Biol.* 64 371–386 10.1007/s11103-007-9159-6 17453154

[B31] Kirigwi F. M. Van Ginkel M. Brown-Guedira G. Gill B. S. Paulsen G. M. Fritz A. K. (2007). Markers associated with a QTL for grain yield in wheat under drought. *Mol. Breed.* 20 401–413 10.1007/s11032-007-9100-3

[B32] Latiri K. Lhomme J. P. Lawlor D. (2013). Grains filling of durum wheat through assimilate remobilisation under semi-arid conditions. *Exp. Agric.* 49 197–211 10.1017/S0014479712001238

[B33] Lawlor D. W. (2002a). Carbon and nitrogen assimilation in relation to yield: mechanisms are the key to understanding production systems. *J. Exp. Bot.* 53 773–787 10.1093/jexbot/53.370.773 11912221

[B34] Lawlor D. W. (2002b). Limitation to photosynthesis in water-stressed leaves: stomata vs. metabolism and the role of ATP. *Ann. Bot.* 89 871–885 10.1093/aob/mcf110 12102513 PMC4233810

[B35] Lawlor D. W. (2013). Genetic engineering to improve plant performance under drought: physiological evaluation of achievements, limitations, and possibilities. *J. Exp. Bot.* 64 83–108 10.1093/jxb/ers326 23162116

[B36] Lawlor D. W. Tezara W. (2009). Causes of decreased photosynthetic rate and metabolic capacity in water deficient leaf cells: a critical evaluation of mechanisms and integration of processes. *Ann. Bot.* 103 561–579 10.1093/aob/mcn244 19155221 PMC2707350

[B37] Lee S-B. Kwon H-B. Kwon S-J. Park S-C. Jeong M-J. Han S-E. (2003). Accumulation of trehalose within transgenic chloroplasts confers drought tolerance. *Mol. Breed.* 11 1–13 10.1023/A:1022100404542

[B38] Lisso J. Schröder F. Müssig C. (2013). EXO modifies sucrose and trehalose responses and connects the extracellular carbon status to growth. *Front. Plant Sci.* 4:219 10.3389/fpls.2013.00219 PMC3691544 23805150

[B39] Lunn J. E. Delorge I. Figueroa C. M. Van Dijck P. Stitt M. (2014). Trehalose metabolism in plants. *Plant J.* 79 544–567 10.1111/tjp.12509 24645920

[B40] Maccaferri M. Sanguineti M. C. Corneti S. Araus J. L. Ben Salem M. Bort J. (2008). Quantitative trait loci for grain yield and adaptation of durum wheat (*Triticum durum* Desf.) across a wide range of water availability. *Genetics* 178 489–511 299–308 10.1534/genetics.107.077297 18202390 PMC2206097

[B41] Martínez-Barajas E. Delatte T. Schluepmann H. de Jong G. J. Somsen G. W. Nunes C. (2011). Wheat grain development is characterized by remarkable trehalose 6-phosphate accumulation pregrain filling: tissue distribution and relationship to SNF1-related protein kinase1 activity. *Plant Physiol.* 156 373–381 10.1104/pp.111.174524 21402798 PMC3091070

[B42] Miralles D. J. Slafer G. A. (2007). Sink limitations to yield in wheat: how could it be reduced? *J. Agric. Sci.* 145 139–149 10.1017/S0021859607006752

[B43] Mitchell R. A. C. Gibbard C. L. Mitchell V. J. Lawlor D. W. (1996). Effects of shading in different developmental phases on biomass and grain yield of winter wheat at ambient and elevated CO_2_. *Plant Cell Environ.* 19 651–621 10.1111/j.1365-3040.1996.tb00396.x

[B44] Mitchell R. A. C. Mitchell V. J. Driscoll S. P. Franklin J. Lawlor D. W. (1993). Effects of increased CO_2_ concentration and temperature on growth and yield of winter wheat at two levels of nitrogen application. *Plant Cell Environ.* 16 521–529 10.1111/j.1365-3040.1993.tb00899.x

[B45] Nunes C. O’Hara L. E. Primavesi L. F. Delatte T. L. Schluepmann H. Somsen G. W. (2013a). The Trehalose 6-Phosphate/SnRK1 signaling pathway primes growth recovery following relief of sink limitation. *Plant Physiol.* 162 1720–1732 10.1104/pp.113.220657 23735508 PMC3707538

[B46] Nunes C. Primavesi L. F. Patel M. K. Martinez-Barajas E. Powers S. J. Sagar R. (2013b). Inhibition of SnRK1 by metabolites: tissue-dependent effects and cooperative inhibition by glucose 1-phosphate in combination with trehalose 6-phosphate. *Plant Physiol. Biochem.* 63 89–98 10.1016/j.plaphy.2012.11.011 23257075

[B47] O’Hara L. E. Paul M. J. Wingler A. (2013). How do sugars regulate plant growth and development? New insight into the role of trehalose-6-phosphate. *Mol. Plant* 6 261–274 10.1093/mp/sss120 23100484

[B48] Patrick J. W. Oﬄer C. E. (2001). Compartmentation of transport and transfer events in developing seeds. *J. Exp. Bot.* 52 551–564 10.1093/jexbot/52.356.551 11373304

[B49] Paul M. (2007). Trehalose 6-phosphate. *Curr. Opin. Plant Biol.* 10 1–7 10.1016/j.pbi.2007.04.001 17434789

[B50] Paul M. J. Primavesi L. F. Jhurreea D. Zhang Y. (2008). Trehalose metabolism and signalling. *Annu. Rev. Plant Biol.* 59 417–441 10.1146/annurev.arplant.59.032607.092945 18257709

[B51] Paul M. J. Jhurreea D. Zhang Y. Primavesi L. F. Delatte T. Schluepmann H. (2010). Up-regulation of biosynthetic processes associated with growth by trehalose 6-phosphate. *Plant Signal. Behav.* 5 386–392 10.4161/psb.5.4.10792 20139731 PMC2958589

[B52] Pellny T. K. Ghannoum O. Conroy J. P. Schluepmann H. Smeekens S. Andralojc J. (2004). Genetic modification of photosynthesis with *E. coli* genes for trehalose synthesis. *Plant Biotechnol. J.* 2 71–82 10.1111/j.1467-7652.2004.00053.x 17166144

[B53] Primavesi L. F. Paul M. J. Schluepmann H. (2011). Growth arrest by trehalose-6-phosphate: an astonishing case of primary metabolite control over growth by way of the SnRK1 signaling pathway. *Plant Physiol.* 157 160–174 10.1104/pp.111.180422 21753116 PMC3165867

[B54] Quarrie S. A. Pekic Quarrie S. Radosevic R. Rancic D. Kaminska A. Barnes J. D. (2006). Dissecting a wheat QTL for yield present in a range of environments: from the QTL to candidate genes. *J. Exp. Bot.* 57 2627–2637 10.1093/jxb/erl026 16831847

[B55] Raines C. A. (2011). Increasing photosynthetic carbon assimilation in C3 plants to improve crop yield: current and future strategies. *Plant Physiol.* 155 36–42 10.1104/pp.110.168559 21071599 PMC3075778

[B56] Reynolds M. Bonnett D. Chapman S. C. Furbank R. T. Manés Y. Mather D. E. (2011). Raising yield potential of wheat. I. Overview of a consortium approach and breeding strategies. *J. Exp. Bot.* 62 439–452 10.1093/jxb/erq311 20952629

[B57] Reynolds M. P. Rajaram S. McNab A. (eds) (1996). *Increasing Yield Potential in Wheat: Breaking the Barriers.* Mexico, DE: CIMMYT

[B58] Richards R. A. Rebetzke G. J. Watt M. Condon A. G. Spielmeyer W. Dolferus R. (2010). Breeding for improved water productivity in temperate cereals: phenotyping, quantitative trait loci, markers and the selection environment. *Funct. Plant Biol.* 37 85–97 10.1071/FP09219

[B59] Satoh-Nagasawa N. Nagasawa N. Malcomber S. Sakai H. Jackson D. (2006). A trehalose metabolic enzyme controls inflorescence architecture in maize. *Nature* 441 227–230 10.1038/nature04725 16688177

[B60] Schluepmann H. Pellny T. van Dijken A. Smeekens S. Paul M. (2003). Trehalose 6-phosphate is indispensable for carbohydrate utilization and growth in *Arabidopsis thaliana*. *Proc. Natl. Acad. Sci. U.S.A.* 100 6849–6854 10.1073/pnas.1132018100 12748379 PMC164535

[B61] Slafer G. A. (2007). “Physiological determination of major wheat yield components,” in *Wheat Production in Stressed Environments* eds Buck H. T. Nisi J. E. Salomón N. (Dordrecht: Springer) 557–565 10.1007/1-4020-5497-1_68

[B62] Slafer G. A. (2010). *Physiology of crop yield. Opportunities to improve productivity of crop plants*. IAEA – Cursos Regional Metedos de Evaluation de Mutantes, Feb 1–5 2010 Available at: http://ciat.cgiar.org/wp-content/uploads/2012/11/2010_02_04_G_Slafer.pdf

[B63] Slafer G. A. Araus J. L. (2007). “Physiological traits for improving wheat yield under a wide range of environments,” in *Scale and Complexity in Plant Systems Research* (Wageningen UR Frontis Series, Vol. 21) eds Spiertz J. H. J. Struik P. C. van Laar H. H. (Dordrecht: Springer) 147–156

[B64] Slafer G. A. Kantolic A. G. Appendino M. L. Miralles D. J. Savin R. (2009). “Crop development: genetic control, environmental modulation and relevant for genetic improvement of crop yield,” in *Crop Physiology: Applications for Genetic Improvement and Agronomy* eds Sadras V. O. Calderini D. F. (Amsterdam: Elsevier) 277–308

[B65] Stiller I. Dulai S. Kondrák M. Tarnai R. Szabó L. Toldi O. (2008). Effects of drought on water content and photosynthetic parameters in potato plants expressing the trehalose-6-phosphate synthase gene of *Saccharomyces cerevisiae*. *Planta* 227 299–309 10.1007/s00425-007-0617-9 17828416

[B66] van den Ende W. (2013). Multifunctional fructans and raffinose family oligosaccharides. *Front. Plant Sci.* 4:427 10.3389/fpls.2013.00247 PMC3713406 23882273

[B67] van Dijken A. J. H. Schluepmann H. Smeekens S. C. M. (2004). Arabidopsis trehalose-6-phosphate synthase 1 is essential for normal vegetative growth and transition to flowering. *Plant Physiol.* 135 969–977 10.1104/pp.104.039743 15181208 PMC514131

[B68] Verma V. Foulkes M. J. Worland A. J. Sylvester-Bradley R. Caligari P. D. S. and Snape J. W. (2004). Mapping quantitative trait loci for flag leaf senescence as a yield determinant in winter wheat under optimal and drought-stressed environments. *Euphytica* 135 255–263 10.1023/B:EUPH.0000013255.31618.14

[B69] Williams T. C. R. Poolman M. G. Howden A. J. M. Schwarzlander M. Fell D. A. Ratcliffe G. R. (2010). A genome-scale metabolic model accurately predicts fluxes in central carbon metabolism under stress conditions. *Plant Physiol.* 154 311–323 10.1104/pp.110.158535 20605915 PMC2938150

[B70] Wingler A. Delatte T. L. O’Hara L. E. Primavesi L. F. Jhurreea D. Paul M. J. (2012). Trehalose 6-Phosphate is required for the onset of leaf senescence associated with high carbon availability. *Plant Physiol.* 158 1241–1251 10.1104/pp.111.191908 22247267 PMC3291265

[B71] Yadav U. P. Ivakov A. Feil R. Duan G. Y. Walther D. Giavalisco P. (2014). The sucrose–trehalose 6-phosphate (Tre6P) nexus: specificity and mechanisms of sucrose signalling by Tre6P. *J. Exp. Bot.* 65 1051–1068 10.1093/jxb/ert457 24420566 PMC3935566

[B72] Yeo E. T. Kwon H. B. Han S. E. Lee J. T. Ryu J. C. Byun M. O. (2000). Genetic engineering of drought resistant potato plants by introduction of the trehalose-6-phosphate synthase (TPS1) gene from *Sacharomyces cerevisae*. *Mol. Cells* 10 263–268 10901163

[B73] Zhang Y. Primavesi L. F. Jhurreea D. Andralojc P. J. Mitchell R. A. C. Powers S. J. (2009). Inhibition of SNF1 related protein kinase1 activity and regulation of metabolic pathways by trehalose-6-phosphate. *Plant Physiol.* 149 1860–1871 10.1104/pp.108.133934 19193861 PMC2663748

